# Memory complaints and depressive symptoms over time: a construct-level replication analysis

**DOI:** 10.1186/s12877-020-1451-1

**Published:** 2020-03-02

**Authors:** Jacqueline Mogle, Nikki L. Hill, Sakshi Bhargava, Tyler Reed Bell, Iris Bhang

**Affiliations:** 1 College of Health and Human Development, Pennsylvania State University, 320D Biobehavioral Health Building, University Park, Pennsylvania, PA 16802 USA; 2 College of Nursing, Pennsylvania State University, 201 Nursing Sciences Building, University Park, Pennsylvania, PA 16802 USA

**Keywords:** Memory complaints, Depressive symptoms, Cognitive decline, Older adults, Replication analysis

## Abstract

**Background:**

Memory complaints and depressive symptoms are frequently associated in older adults and both serve as potential indicators of future cognitive decline. However, the temporal ordering of the development of these two symptoms remains unclear. The goal of the current study was to examine concurrent and temporal relationships between memory complaints and depressive symptoms in older adults.

**Methods:**

Data were drawn from two longitudinal, nationally representative datasets and included cognitively intact older adults aged 65 and over. The datasets in the current study were from the National Health and Aging Trends Study (NHATS) and the Health and Retirement Study (HRS). Using an integrative analytic framework, we tested bidirectional temporal relationships between memory complaints (memory ratings and perceived memory decline) and depressive symptoms over 6 to 9 years of data in over 5000 older adults across these two samples.

**Results:**

Across both datasets, perceived memory decline predicted future depressive symptoms whereas memory ratings did not. Additionally, results showed that at times when depressive symptoms tended to be higher, memory complaints were also higher, but depressive symptoms did not predict future memory complaints. One finding that was inconsistent across datasets was memory ratings predicting depressive symptoms. After accounting for covariates, this relationship was only significant in one dataset.

**Conclusions:**

Cognitively intact older adults who report memory decline may be at risk for developing depressive symptoms in the future. Furthermore, our findings highlight the importance of using immediate replication of results across datasets to determine the generalizability of conclusions.

## Background

Depressive symptoms and changes in memory functioning frequently co-occur in late life, complicating the distinction among symptoms due to mood disorders, preclinical or early dementia, and other geriatric syndromes. This distinction is particularly important for assessment of cognitive decline risk since both depressive symptoms and memory complaints (where an individual perceives a change in memory functioning but performs normally on objective tests) are associated with poor cognitive outcomes in older adults. Memory complaints may reflect early cognitive decline or normal aging, but they are also clustered among depressive symptoms used for diagnosis [1]. Indeed, memory complaints frequently have stronger associations with depressive symptoms than with objective cognition [2–4]. However, memory complaints uniquely predict a substantially increased risk of cognitive decline and dementia after controlling for depressive symptoms [5–7]. Further, in a recent study, depression and memory complaints were independently associated with the risk of mild cognitive impairment and dementia [8]. This suggests memory complaints reflect a related but separable source of variation in the risk for cognitive decline. Given their co-occurrence and unique predictive utility, it is important to understand how memory complaints and depressive symptoms develop over time among older adults: specifically, whether memory complaints tend to precede depressive symptoms, or vice versa. The current paper applies an integrative analytic framework using two nationally representative datasets to examine the longitudinal bidirectional associations of memory complaints and depressive symptoms to understand these temporal associations.

Previous longitudinal studies have primarily examined temporal relationships between depressive symptoms and memory complaints in a single direction. For example, when examining concurrent within-person relationships, Hülür, Hertzog, Pearman, Ram, and Gerstorf [9] focused on depressive symptoms predicting memory complaints and found that on occasions when an individual reported more depressive symptoms, they tended to report more memory complaints. Testing specifically whether memory complaints preceded depressive symptoms, multiple studies have identified that individuals reporting memory problems at baseline are at risk for developing clinical levels of depressive symptoms over four [10] and 10 [11] years of follow up [12]. However, it remains unclear whether the opposite relationship would hold in these studies. That is, when an individual is experiencing more depressive symptoms, are they more likely to report memory complaints at the next assessment. This is particularly important as it could identify the temporal relationships among depressive symptoms and memory complaints, indicating which type of symptom tends to appear first.

Another limitation of current work examining the relationships among memory complaints and depressive symptoms is the wide variability in approaches to assessing memory complaints [13]. Participants may be asked to rate their current memory performance, compare their memory performance to that of their peers, or reflect on whether their memory has declined over time. Each of these questions requires older adults to construct different judgements about their memory functioning that seem somewhat contradictory [14]. For example, an individual might perceive their memory as declining (poorer than the previous year) while still believing their current memory functioning to be good in general and better than others their age. This can be further complicated by the time periods across which individuals are asked to judge changes in memory performance, which can vary widely from change over the past year to change over the past 10 years. Recent studies examining how older adults interpret questions about their memory performance indicate that these different types of questions indeed assess substantively different aspects of the experience of memory problems [15, 16]. Therefore, it is important to consider how items assessing memory complaints may be differentially related to current and future depressive symptoms.

Integrative analysis using comparable assessments of memory complaints can deliver more reproducible conclusions through examination of research questions across multiple datasets simultaneously [17]. Given the previous lack of research examining autoregressive bidirectional temporal relationships among depressive symptoms and memory complaints, this approach provides an immediate replication that can aid in building scientific evidence for these temporal relationships. Our primary goal in the current analyses was to expand on previous work by examining bidirectional temporal associations among memory complaints and depressive symptoms using equivalent modeling (e.g., similar covariates and model specifications) across the datasets. This allowed us to address our research question of whether higher depressive symptoms are consistently related to future reports of memory complaints or vice versa. Importantly, we examined whether the temporal sequencing of these symptoms depended on the type of memory complaint assessment used (memory rating and perceived memory decline). We capitalized on autoregressive modeling of depressive symptoms and memory complaints to determine whether we could identify temporal sequencing of symptoms in older adults across two independent samples, both longitudinal datasets that used similar memory complaint assessments.

## Methods

### Participants

The study samples were drawn from the National Health and Aging Trends Study (NHATS) and the Health and Retirement Study (HRS). NHATS employs a longitudinal design, has a nationally representative sample of adults age 65 and older who are Medicare beneficiaries in the United States, and oversamples Black older adults and individuals at older ages [18]. The study began in 2011 and is funded by the National Institute on Aging (NIA) and led by the Johns Hopkins University Bloomberg School of Public Health. Face-to-face interviews are conducted annually and collect data on topics such as physical and cognitive capacity and participation in valued activities. HRS is a longitudinal, nationally representative sample of adults older than 50 in the United States, with an oversampling of Blacks and Hispanics [19]. HRS is funded by NIA and is led by the Institute for Social Research at the University of Michigan. Started in 1992, HRS collects participants’ data biennially in mainly face-to-face or telephone interviews. The four main types of data are: income and wealth; health, cognition, and use of healthcare services; work and retirement; and family connections. Both NHATS and HRS undergo periodic cohort replenishment.

#### NHATS

The current study used data from NHATS waves 1 to 6, collected annually. To be included in the current study, older adults must have completed self-reports of memory complaints, have no diagnosis of Alzheimer’s disease (AD) or mild cognitive impairment (MCI), should have demonstrated good or fair understanding of questions and didn’t need help answering, and completed interviews in English. Following Kasper, Freedman, & Spillman’s [20] criteria for probable cognitive impairment, an algorithm additionally detected and removed individuals who performed < 1.5 SD on two or more cognitive domains (tasks described in Kasper et al. [20]). At wave 1, 8245 older adults participated in NHATS; however, only 3257 older adults met the eligibility criteria described above. In addition, 160 older adults who identified as Hispanic, Other, or didn’t identify with any race/ethnicity were excluded from analysis as the small number of individuals would result in unbalanced racial groups that would make it difficult to draw conclusions about race differences in effects. Therefore, the current study included 3097 older adults from cohort 1. Participants in cohort 1 had on average 3 waves of data (*M* = 3.16, *SD* = 1.66) with 51.89% of the sample completing all 6 waves of data (*n* = 1607). At wave 5, NHATS replenished the sample with cohort 2 and 4182 older adults were added, of which 2081 met the criteria to be included in the current study. Similar to the first NHATS cohort, 109 older adults who identified as Hispanic, Other, or didn’t identify with any race/ethnicity were excluded from analysis as the small number of individuals would result in unbalanced racial groups. Therefore, a total of 1972 older adults were included from wave 5 and 92.64% of these older adults participated at wave 6 (*n* = 1827). Waves were restructured for the replenished sample such that wave 5 represented time 1 and wave 6 represented time 2. For purposes of the current study, approximately 60% of participants had at least 4 waves of follow up data. Overall, the current study included 5069 older adults (78.77% White; 21.23% Black; 63.64% Female) at time 1. See Table 1 for sample description.

**Table 1:** NHATS and HRS Sample Description at Baseline

	NHATS (*n* = 5069)	HRS (*n* = 5505)
	n (%)	n (%)
Sex		
Male	1843 (36.36)	1939 (35.22)
Female	3226 (63.64)	3566 (64.78)
Race		
White	3993 (78.77)	4935 (89.65)
Black	1076 (21.23)	570 (10.35)
Age		
65–69	1437 (28.35)	307 (5.58)
70–74	1295 (25.55)	1591 (28.90)
75–79	983 (19.29)	1750 (31.79)
80–84	790 (15.58)	1122 (20.38)
85–89	375 (7.40)	549 (9.97)
90+	189 (3.73)	186 (3.38)
Education		
Less than HS	660 (13.03)	2096 (38.07)
High School	1441 (28.44)	2604 (47.30)
Greater than HS	2966 (58.54)	805 (14.62)
Income		
Less than $15,000	2624 (51.77)	2015 (36.60)
$15,000 – $30,000	687 (13.55)	1839 (33.41)
$30,001 – $60,000	859 (16.95)	1102 (20.02)
$60,000 and above	899 (17.74)	549 (9.97)
Marital Status		
Married/Living with a partner	2436 (48.08)	2633 (47.84)
Separated/Divorced/ Widowed/Never married	2631 (51.92)	2872 (52.16)

#### HRS

Data from HRS waves 3 to 12 were used from the AHEAD cohort as they contained the memory complaint items that were most similar, and comparable to, NHATS items. These data were collected biennially. Eligibility included completing self-reports of memory complaints, no diagnosis of AD or MCI, and participants did not need proxy-assistance for reporting. Similar to NHATS, an algorithm flagged individuals for probable cognitive impairment if they performed < 1.5 SD on two or more cognitive domains (tasks described in McArdle, Smith, & Willis [21]). In the AHEAD cohort, 6879 individuals 65 years and older participated in HRS at wave 3. Of these, 5597 met the eligibility criteria described above. Similar to our decisions with the NHATS sample, 92 Hispanic individuals were removed so as to allow us to assess comparisons across races. Thus, 5505 participants from the AHEAD cohort were available for analysis at time 1 (89.65% White; 10.35% Black; 64.78% Female). See Table 1 for sample description. Participants, on average, completed 3 waves of data (*M* = 3.55, *SD* = 2.35) and 5% of the sample completed data at wave 12 in the current study (*n* = 323). Attrition rate in the HRS sample is consistent with attrition rates in other studies with older participants that show a loss of 15–20% of the sample at each wave mainly due to aging of the participants [22, 23]. For the purposes of the current study, approximately 50% of participants had at least four waves of follow up data.

### Measures

Descriptive statistics for the study measures described below are provided in Table 1.

#### Depressive symptoms

In NHATS, depressive symptoms were measured using the Patient Health Questionnaire-2 (PHQ2), a validated screening measure for depression [24]. Participants responded to two questions (“*Over the past month, how often have you had little interest or pleasure in doing things””* and “… *felt down, depressed, or hopeless?”)* on a four-point scale: 1 = not at all, 2 = several days, 3 = more than half the days, or 4 = nearly every day. Scores ranged from 2 to 8 with higher scores indicating more depressive symptoms [24, 25]. At baseline, this measure had a reliability of 0.57. The reliability for the measure was higher at .80 [26] when considering most participants had at least four repeated observations.

In HRS, depressive symptoms were measured with an adapted version of the Center for Epidemiological Studies Depression Scale (CES-D [27]). Participants responded to eight items that asked about their feelings over the past week (“was depressed”, “everything was an effort”, “sleep was restless”, “was happy”, “felt lonely”, “enjoyed life”, “felt sad”, and “could not get going”)*.* Participants responded on a dichotomous scale (0 = no; 1 = yes). Two items (“was happy” and “enjoyed life”) were reverse coded. Scores ranged from 0 to 8. Higher scores indicated more depressive symptoms [28]. At baseline, this measure had a reliability of 0.74 and a reliability of .82 when accounting for repeated observations [26].

#### Memory complaints

Both NHATS and HRS included only two items that assessed memory complaints at each wave. In NHATS, memory ratings were assessed with the item, “*First, how would you rate your memory at the present time?,*” with response selections on a five-point scale: 1 = excellent, 2 = very good, 3 = good, 4 = fair, and 5 = poor. Perceived memory decline was assessed with the item, “*Compared to one year ago, would you say your memory is much better now, better now, about the same, worse now, or much worse now than it was then?*” with response options on a five-point scale: 1 = much better, 2 = better, 3 = same, 4 = worse, and 5 = much worse. Due to low variability, response options for this variable were re-coded to create a binary variable (0 = much better/better/same; 1 = worse/much worse).

In HRS, memory complaints were measured with two similar items. Memory ratings were assessed with the item, “*How would you rate your memory at the present time?*” with response options on a five-point scale: 1 = excellent, 2 = very good, 3 = good, 4 = fair, and 5 = poor. Perceived memory decline was assessed with the item, “*Compared with (previous wave/two years ago), would you say your memory is better now, about the same, or worse than it was then?*” with response options on a three-point scale: 1 = better, 2 = same, and 3 = worse. Due to low variability, response options for this variable were re-coded to create a binary variable (0 = better/same; 1 = worse).

#### Covariates

Several demographic factors have been shown to correspond to either memory complaints or depressive symptoms including sex, age, education, and income [29, 30]; therefore, each of these were included as covariates in our integrative analyses. To have consistent measures of covariates across NHATS and HRS, the following variables were recoded as needed. Participants’ sex (1 = male, 2 = female), race (1 = White/non-Hispanic, 2 = Black/non-Hispanic), age, education, income, and marital status were included as covariates. In NHATS, age is reported as a categorical variable (1 = 65–69; 2 = 70–74; 3 = 75–79; 4 = 80–84; 5 = 85–89; 6 = 90+ years); therefore, the HRS age variable was recoded to be consistent. Education was coded to three categories (1 = less than high school, 2 = high school, 3 = more than high school [31] and income was a four category variable (1 = less than $15,000; 2 = $15,000 - $30,000; 3 = $30,001 to $60,000; 4 = $60,000 and above). Marital status was represented as a two-category variable (0 = married/living with a partner; 1 = separated/divorced/widowed/never married).

### Statistical analysis

Prior to examining the proposed research questions, we conducted descriptive analyses to identify any mean differences in memory complaints and depressive symptoms based on participants’ race, age, sex, income, education, and marital status. Additionally, mean differences in age, income, education, and marital status were examined by race and sex. Next, intercorrelations were examined among the key study variables.

To address our primary research questions, multilevel linear modeling (MLM) analyses were performed. MLM allows the testing of longitudinal relationships among variables when there are uneven amounts of follow-up for participants as well as the separation of within-person effects (i.e., how variables change within an individual over time) from differences across individuals. Depressive symptoms and memory ratings were treated as continuous outcomes and modeled using SAS proc. mixed. Effects from these models are reported as regression coefficients where a one-unit change in the predictor is associated with the amount of change in the outcome indicated by the estimate. Perceived memory decline was a binary variable and was modeled using SAS proc. glimmix using a binary distribution with a logit link. Effects from these models are reported as odds ratios which are the result of exponentiated regression coefficients.

Prior to addressing our research questions, we fit unconditional means models to examine the intraclass correlations and determine the proportion of variance in depressive symptoms, memory ratings, and perceived memory decline that could be explained by individual differences relative to change within an individual over time. Next, unconditional growth models were performed to examine the trajectories of depressive symptoms, memory ratings, and perceived memory decline.

To address our research questions regarding the temporal nature of memory complaints and depressive symptoms, we first examined whether decreases in memory ratings preceded increases in depressive symptoms. Autoregressive models were fit to the data to examine the concurrent and lagged effects of memory ratings on depressive symptoms. To examine the lagged effects, changes in memory ratings from the previous wave (i.e., time *t*-1) were entered as a lagged variable to predict future depressive symptoms (i.e., time *t*). We then examined the reciprocal relationship (i.e., do increases in depressive symptoms tend to precede decreases in memory ratings) with autoregressive models that tested the concurrent and lagged effects of depressive symptoms on memory ratings. We then replicated these models with the perceived memory decline items.

For all models, continuous within-person variables (e.g., memory ratings) were baseline centered when used as predictors. That is, an individual’s baseline value was subtracted from that individual’s values at each wave [32]. Perceived memory decline was entered as a raw variable as the 0 point was meaningful within as well as across individuals. Appropriate reference groups were selected for categorical covariates. Following other aging studies (e.g., Lindenberger & Baltes [33]; Nyberg, Bäckman, Erngrund, Olofsson, & Nilsson [34]), we used a criterion *p*-value of .01 to determine which effects were statistically significant; this guards against inflated Type 1 error given our large sample sizes.

## Results

### Preliminary analyses

Baseline comparisons of key study variables by race, sex, age, marital status, education level, and income are presented in Additional file 1: Table S1 for NHATS and Additional file 2: Table S2 for HRS. Intercorrelations among key study variables are presented in Table 2.

**Table 2:** Inter-correlations among Key Study Variables at Baseline

Variable	1	2	3	4	5	6	HRS *M* (SD)
1. Age (cat.)	–	−.075^**^	−.158^**^	.055^**^	.087^**^	.099^**^	–
2. Education (cat.)	−.097^**^	–	.375^**^	−.122^**^	−.006	−.149^**^	–
3. Income (cat.)	−.119^**^	.211^**^	–	−.062^**^	−.009	−.173^**^	–
4. Memory Self-Rating	.062^**^	−.126^**^	−.110^**^	–	.345^**^	.176^**^	2.947 (0.983)
5. Perceived Memory Decline (cat.)	.018	−.007	−.042^*^	.276^**^	–	.161^**^	–
6. Depressive Symptoms	−.014	−.117^**^	−.084^**^	.232^**^	.159^**^	–	1.405 (1.878)
NHATS *M* (SD)	–	–	–	2.455 (0.910)	–	2.760 (1.212)	

*Note*. NHATs coefficients are shown below the diagonal; HRS coefficients are shown above the diagonal; means and standard deviations only shown for continuous variables. Cat. = categorical variable. Kendall Tau coefficients used for associations with categorical variables, Pearson used for continuous outcomes ^**^p ≤ .001, ^*^p ≤ .01

### Multilevel models

#### Intraclass correlation coefficients (ICCs)

For NHATS, ICCs showed that 49.52% of the variance in depressive symptoms, 61.29% of the variance in memory ratings, and 62.27% of the variance in perceived memory decline were due to differences between persons. For HRS, 47.30% of the variance in depressive symptoms, 48.36% of the variance in memory ratings, and 45.36% of the variance in perceived memory decline were due to differences between persons.

#### Unconditional growth models

Unconditional growth models examined the trajectories of depressive symptoms, memory ratings, and perceived memory decline. For NHATS, on average, reports of depressive symptoms did not change over time (*b* = 0.002, *SE* = 0.004, *p* = .679), whereas reports of poorer memory ratings (*b* = 0.063, *SE* = 0.003, *p* < .001) and perceived memory decline (*OR* = 1.182; 95% CI: 1.134–1.233) increased over time. For HRS, reports of depressive symptoms (*b* = 0.085, *SE* = 0.004, *p* < .001), poorer memory ratings (*b* = 0.050, *SE* = 0.002, *p* < .001), and perceived memory decline (*OR* = 1.169; 95% CI: 1.150–1.189) increased over time.

#### Substantive models

Substantive analyses examined: 1) concurrent associations of memory complaints (memory ratings and perceived memory decline) with depressive symptoms, 2) lagged effects of memory complaints on depressive symptoms, and 3) lagged effects of depressive symptoms on memory complaints. Below we report findings after accounting for the associations of all covariates.

#### Concurrent and lagged effects of memory complaints on depressive symptoms

For NHATS, concurrent (*b* = 0.027, *SE* = 0.013, *p* = 0.048) and lagged effects (*b* = − 0.013, *SE* = 0.016, *p* = .430) of memory ratings on depressive symptoms were not significant. For HRS, on average, memory ratings and depressive symptoms were related concurrently (*b* = 0.179, *SE* = 0.015, *p* < .001) such that, at waves when an individual reported poorer memory ratings, they also reported more depressive symptoms. Like NHATS, HRS memory ratings did not have a positive lagged effect on depressive symptoms (*b* = 0.033, *SE* = 0.016, *p* = .042; see Table 3 for all estimates).

**Table 3:** Results of Multilevel Models Examining Concurrent and Lagged Effects of Memory Self-Report on Depressive Symptoms

	Depressive Symptoms			
	NHATS		HRS	
	Model 1 *b (SE)*	Model 2 *b (SE)*	Model 1 *b (SE)*	Model 2 *b (SE)*
Intercept	2.731^**^ (0.046)	2.705^**^ (0.048)	1.188^**^ (0.128)	0.864 ^**^ (0.128)
Time	0.009 (0.006)	0.002 (0.006)	0.030^**^ (0.005)	0.025^**^ (0.005)
Self-rated Memory_wp_	0.027 (0.013)	–	0.179^**^ (0.015)	–
Self-rated Memory_lag_	− 0.013 (0.016)	–	0.033 (0.016)	–
Perceived 2-year Decline_wp_	–	0.407^**^ (0.032)	–	0.456^**^ (0.029)
Perceived 2-year Decline_lag_	–	0.195^**^ (0.032)	–	0.269^**^ (0.030)
Female (ref = male)	0.146^**^ (0.031)	0.141^**^ (0.031)	0.267^**^ (0.052)	0.251^**^ (0.052)
Education: < HS (ref = HS education)	0.093 (0.050)	0.082 (0.049)	0.288^**^ (0.054)	0.322^**^ (0.053)
Education: > HS (ref = HS education)	−0.206^**^ (0.034)	− 0.216^**^ (0.033)	− 0.338^**^ (0.069)	− 0.388^**^ (0.068)
Age category	− 0.012 (0.228)	− 0.049 (0.222)	0.449 (0.467)	0.394 (0.461)
Black (ref = White)	0.159^**^ (0.038)	0.153^**^ (0.038)	0.079 (0.079)	0.078 (0.078)
Income $15,000 - $30,000 (ref = < $15,000)	0.052 (0.045)	0.051 (0.044)	−0.286^**^ (0.062)	−0.278^**^ (0.061)
Income $30,001–$60,000 (ref = < $15,000)	−0.070 (0.040)	− 0.057 (0.040)	−0.332^**^ (0.075)	− 0.342^**^ (0.074)
Income > $60,000 (ref = < $15,000)	−0.166^**^ (0.041)	− 0.158^**^ (0.040)	−0.399^**^ (0.093)	− 0.416^**^ (0.092)
Single/Widowed (ref = Married/Partnered)	0.071 (0.032)	0.072 (0.032)	0.041 (0.056)	−0.030 (0.056)

*Note.* NHATS and HRS data were analyzed in separate multilevel analyses. Additionally, Models 1 and 2 were analyzed separately. Model 1 examined the association of self-rated memory with depressive symptoms whereas Model 2 examined the association of perceived 2-year memory decline with depressive symptoms. Results from analyses with NHATS and HRS data are presented together for ease of comparison. All models were analyzed separately. *HS* = High School; WP = baseline within person. Lag = variable information used from one-year prior. ^**^p ≤ .001, ^*^p ≤ .01

For both NHATS and HRS, on average, perceived memory decline and depressive symptoms shared a positive concurrent association such that, at waves when individuals reported memory decline, they also reported more depressive symptoms (NHATS: *b* = 0.407, *SE* = 0.032, *p* < .001; HRS: *b* = 0.456, *SE* = 0.029, *p* < .001). Additionally, perceived memory decline had a positive lagged effect on depressive symptoms (NHATS: *b* = 0.195, *SE* = 0.032, *p* < .001; HRS: *b* = 0.269, *SE* = 0.030, *p* < .001) such that, when individuals reported memory decline at a given wave, they reported more depressive symptoms at the subsequent wave (see Table 3).

#### Concurrent and lagged effect of depressive symptoms on memory complaints

For both NHATS and HRS, on average, depressive symptoms shared a positive concurrent association with memory ratings such that, at waves when individuals reported more depressive symptoms, they also rated their memory as being poorer (NHATS: *b* = 0.021, *SE* = 0.006, *p* < .001; HRS: *b* = 0.022, *SE* = 0.004, *p* < .001). However, depressive symptoms did not have a lagged effect on memory ratings (NHATS: *b* = 0.003, *SD* = 0.007, *p* = .706; HRS: *b* = 0.009, *SE* = 0.004, *p* = .029), such that reports of depressive symptoms at any given wave did not predict memory ratings at subsequent waves above chance (see Table 4).

**Table 4:** Results of Multilevel Models Examining Concurrent and Lagged Effects of Depressive Symptoms on Memory Self-Report

	Self-rated Memory		Perceived Memory Decline	
	Model 1		Model 2	
	NHATS *b (SE)*	HRS *b (SE)*	NHATS *OR (95%CI)*	HRS *OR (95%CI)*
Intercept	2.552^**^ (0.019)	2.902^**^ (0.061)	–	**–**
Time	0.059^**^ (0.004)	0.048^**^ (0.003)	1.209^**^ (1.143–1.279)	1.144^**^ (1.120–1.169)
Depressive Symptoms_wp_	0.021^**^ (0.006)	0.022^**^ (0.004)	1.286^**^ (1.189–1.390)	1.145^**^ (1.115–1.177)
Depressive Symptoms_lag_	0.003 (0.007)	0.009 (0.004)	1.040 (0.948–1.142)	1.012 (0.982–1.043)
Female (ref = male)	−0.006 (0.025)	−.0.122^**^ (0.026)	1.131 (0.864–1.479)	0.885 (0.751–1.044)
Education: <HS (ref = HS education)	0.056 (0.040)	0.138^**^ (0.027)	1.377 (0.906–2.093)	0.854 (0.720–1.013)
Education: > HS (ref = HS education)	−0.193^**^ (0.028)	− 0.143^**^ (0.035)	1.368 (1.019–1.836)	1.296 (1.048–1.602)
Age category	−0.096 (0.182)	− 0.356 (0.235)	1.291 (0.203–8.196)	1.200 (0.269–5.350)
Black (ref = White)	0.193^**^ (0.031)	0.098 (0.040)	0.984 (0.709–1.367)	1.131 (0.880–1.453)
Income $15,000 - $30,000 (ref = < $15,000)	0.001 (0.036)	−0.032 (0.030)	1.051 (0.727–1.521)	0.856 (0.702–1.043)
Income $30,001–$60,000 (ref = < $15,000)	−0.072 (0.032)	− 0.071 (0.038)	0.654 (0.459–0.932)	0.976 (0.771–1.234)
Income > $60,000 (ref = < $15,000)	−0.143^**^ (0.033)	− 0.068 (0.047)	0.792 (0.558–1.124)	1.024 (0.765–1.370)
Single/Widowed (ref = Married/Partnered)	0.023 (0.026)	−0.064 (0.028)	1.021 (0.777–1.343)	0.831 (0.696–0.993)

*Note.* HRS and NHATS datasets were analyzed in separate multilevel analyses. Additionally, the association of depressive symptoms (predictor) with self-rated memory (Model 1) and perceived 2-year memory decline (Model 2) were analyzed separately. Results from analyses with NHATS and HRS data are presented together for ease of comparison. All models were analyzed separately. HS = High School; WP = baseline within person. ^**^p ≤ .001. ^*^p ≤ .01

For both NHATS and HRS, on average, depressive symptoms shared a positive concurrent association with perceived memory decline (NHATS: *OR* = 1.286, 95% CI: 1.189–1.390; HRS: *OR* = 1.145, 95% CI: 1.115–1.177) such that, at waves when individuals reported memory decline, they were also likely to report depressive symptoms. Depressive symptoms did not have a lagged effect on perceived memory decline (NHATS: *OR*: 1.040, 95% CI: 0.948–1.142; HRS: *OR* = 1.012, 95% CI: 0.982–1.043; see Table 4).

## Discussion

The current study applied an integrative analytic framework to better understand temporal associations between depressive symptoms and two types of memory complaints (memory ratings and perceived memory decline) in older adults. Importantly, we found that perceived memory decline was associated with future depressive symptoms over a one- to two-year period consistently across datasets, providing greater confidence in this temporal sequencing of symptoms. In testing the alternative temporal association, depressive symptoms were not associated with future memory complaints (i.e., memory ratings or perceived memory decline) over these same time intervals. We discuss the implications of each of these findings below.

Perceived memory decline, not current memory ratings, was associated with future depressive symptoms across both datasets. This finding suggests that, on average, perceptions of memory *decline* rather than perceptions of *current memory functioning* may precipitate increases in depressive symptoms for older adults. Some older adults who perceive memory declines may be experiencing other aspects of declining function with age (e.g., physical health) that, together with declines in memory functioning, lead to worsening emotional well-being [35]. In contrast, other older adults who perceive declines may be concerned about the meaning of those declines such as whether they are at risk for AD or other dementias, which may in turn lead to increased depressive symptoms [36]. The mechanism that relates perceived memory declines and depressive symptoms likely varies across individuals. Older adults who perceive declines in their memory functioning and experience more depressive symptoms in reaction to this perception may be at increased risk for clinical levels of depression in later life. Identifying those older adults who perceive their memory as declining could help clinicians target this group with interventions intended to normalize the changes in memory that are expected due to aging, and possibly prevent clinically meaningful levels of depressive symptoms. As clinical depression is a risk factor for cognitive decline, AD, and dementia more broadly [37], intervening earlier could help individuals maintain healthy cognitive functioning as they age.

Depressive symptoms were consistently related to concurrent levels of memory complaints across datasets but were unrelated to future memory complaints in these samples. These results followed a pattern identified in previous research on individual differences in which individuals who are depressed are more likely to report problems with their memory [38, 39]. Memory complaints may be among the varied symptoms individuals experiencing negative mood are more likely to report [40, 41]. Alternatively, individuals with depressive symptomatology may be experiencing problems with their memory and attention since impairments in these cognitive domains are frequently associated with depression [42, 43]. However, individual differences in depressive symptoms and memory complaints provide little information about *when* these symptoms develop and whether one symptom tends to precede the other. Importantly, our results indicate that depressive symptoms did not predict future reports of memory complaints in these samples, indicating a lack of evidence that depressive symptoms are the primary driver of the development or worsening of memory complaints over time. Our findings suggest that perceived memory declines, and not ratings of current memory functioning, are more sensitive indicators of risk for future depressive symptoms.

Integrative analysis was valuable for examining these temporal relationships across datasets using systematic methods. By coordinating our analyses, we were able to build stronger scientific evidence that perceived memory decline is an important indicator of future changes in psychological well-being that generalizes beyond a single sample in one set of analyses. Additionally, the integrative approach allows us to ensure that inconsistencies across results were not due to differences in how covariates were coded or how predictors of interest were entered into the model [17]. Another advantage in the current study was that depressive symptoms had the same range of scores allowing us to compare results across datasets and identify remarkably similar temporal relationships among depressive symptoms and memory complaints. Through such harmonious modeling and assessment, integrated analysis revealed replicable links between perceived memory decline and depressive symptoms in older adults.

The current study did have important limitations. As a secondary data analysis, both datasets were collected for other purposes and the assessments included were restricted by the availability of parent study data. This means that the assessments of depressive symptoms were brief and not appropriate for making clinical distinctions among participants. Additionally, the differences in the depression measure across studies could explain some of the lack of relationships observed in NHATS. Similarly, single items were used to assess each type of memory complaint, limiting their reliability. In spite of these weaknesses, both assessments are face valid and captured meaningful variability in the current samples. Another limitation was the use of community samples. Although these samples are more generalizable to the population at large, they are restricted in their range of depressive symptoms and memory complaints. A clinic-based sample of older adults would likely express more variation in these measures which might result in different temporal associations for that subgroup of older adults. Finally, we removed Hispanic participants as they were not well-represented in the current samples. Individuals from diverse ethnic backgrounds are frequently underrepresented in these datasets and including them in group comparisons risks drawing invalid conclusions based on statistical tests [44]. With markedly unbalanced cell sizes, such comparisons violate statistical assumptions (e.g., homogeneity of variance). Future work is needed to increase the representation of these groups in large national samples, as well as empirical work that focuses on these specific subgroups to begin building the evidence for psychological processes across racial and ethnic groups.

Our findings hold potential practice implications, particularly for the assessment of memory complaints and subclinical depression. The differential diagnosis of early dementia and late-life onset depression is a known clinical challenge when patients present with combinations of cognitive, affective, and behavioral symptoms [45]. Cognitive screening measures frequently used in clinical settings, such as the Mini-mental state examination (MMSE [46];), lack sensitivity for identifying milder cognitive deficits and do not discriminate cognitive symptoms of depression from cognitive impairment due to other causes [47]. Since self-reported memory problems may be the earliest symptomatic indicator of AD when cognitive testing is normal [48, 49], and depressive symptoms are known to co-occur with these reports [9, 50], identifying the most common trajectory of these overlapping symptoms is important for guiding early intervention. Asking older adults specifically about their perception of memory decline may better identify those at higher risk for subsequent depressive symptoms; furthermore, addressing concerns about memory decline may help prevent future depression in some older adults.

## Conclusions

The current study took an integrative analytic approach to better understand the concurrent and temporal relationships between memory complaints and depressive symptoms among cognitively intact older adults. Results showed that depressive symptoms tend to co-occur with poorer memory ratings and perceived memory decline in older adults; however, depressive symptoms were not predictive of future memory complaints. In contrast, older adults’ perceived memory decline did predict future depressive symptoms. These findings were replicated across two nationally representative datasets and thus, provide strong evidence that perceived memory decline is an important predictor of future depressive symptoms in older adults. Future studies that examine memory complaints in older adults should consider the differential associations between different types of complaints and depressive symptoms.

## Supplementary information

**Additional file 1: Table S1.** NHATS: Baseline Mean Level Differences in Key Study Variables by Participants’ Age, Sex, Education, Race, Income, and Marital Status.

**Additional file 2: Table S2.** HRS: Baseline Mean Level Differences in Key Study Variables by Participants’ Age, Sex, Education, Race, Income, and Marital Status.

## Data Availability

Both of the datasets analyzed during the current study are publicly available: HRS (http://hrsonline.isr.umich.edu/) and NHATS (https://www.nhats.org/).

## Abbreviations

AD: Alzheimer’s disease
CES-D: Center for Epidemiological Studies Depression scale
HRS: Health and Retirement Study
ICCs: Intraclass correlation coefficients
MCI: Mild cognitive impairment
MLM: Multilevel linear modeling
NHATS: National Health and Aging Trends Study
NIA: National Institute on Aging
PHQ2: Patient Health Questionnaire-2

## References

[1] JESSEN FRANK, WIESE BIRGITT, CVETANOVSKA GABRIELA, FUCHS ANGELA, KADUSZKIEWICZ HANNA, KÖLSCH HEIKE, LUCK TOBIAS, MÖSCH EDELGARD, PENTZEK MICHAEL, RIEDEL-HELLER STEFFI G., WERLE JOCHEN, WEYERER SIEGFRIED, ZIMMERMANN THOMAS, MAIER WOLFGANG, BICKEL HORST (2007). Patterns of subjective memory impairment in the elderly: association with memory performance. Psychological Medicine.

[2] Balash Y., Mordechovich M., Shabtai H., Giladi N., Gurevich T., Korczyn A. D. (2012). Subjective memory complaints in elders: depression, anxiety, or cognitive decline?. Acta Neurologica Scandinavica.

[3] Chin Juhee, Oh Kyung Ja, Seo Sang Won, Na Duk L. (2014). Are depressive symptomatology and self-focused attention associated with subjective memory impairment in older adults?. International Psychogeriatrics.

[4] Kim Jae-Min, Stewart Robert, Shin Il-Seon, Choi Sung-Ku, Yoon Jin-Sang (2003). Subjective Memory Impairment, Cognitive Function and Depression – A Community Study in Older Koreans. Dementia and Geriatric Cognitive Disorders.

[5] Jessen F, Wiese B, Bachmann C, Eifflaender-Gorfer S, Haller F, Kölsch H, et al. Prediction of dementia by subjective memory impairment: effects of severity and temporal association with cognitive impairment. Arch Gen Psychiatry. 2010. 10.1001/archgenpsychiatry.2010.30. 10.1001/archgenpsychiatry.2010.30 20368517

[6] Jonker C, Geerlings MI, Schmand B. Are memory complaints predictive for dementia? A review of clinical and population-based studies. Int J Geriatr Psychiatry. 2000. 10.1002/1099-1166(200011)15:11<983::aid-gps238>3.0.co;2-5. 10.1002/1099-1166(200011)15:11<983::aid-gps238>3.0.co;2-5 11113976

[7] Wang Pei-Ning, Wang Shuu-Jiun, Fuh Jong-Ling, Teng Evelyn Lee, Liu Chia-Yih, Lin Cheng-Huai, Shyu Hann-Yeh, Lu Sbiang-Ru, Chen Chao-Ching, Liu Hsiu-Chih (2000). Subjective Memory Complaint in Relation to Cognitive Performance and Depression: A Longitudinal Study of a Rural Chinese Population. Journal of the American Geriatrics Society.

[8] Liew TM. Depression, subjective cognitive decline, and the risk of neurocognitive disorders. Alzheimers Res Ther. 2019. 10.1186/s13195-019-0527-7. 10.1186/s13195-019-0527-7 PMC6689179 31399132

[9] Hülür Gizem, Hertzog Christopher, Pearman Ann, Ram Nilam, Gerstorf Denis (2014). Longitudinal associations of subjective memory with memory performance and depressive symptoms: Between-person and within-person perspectives. Psychology and Aging.

[10] Heun Reinhard, Hein Sandra (2005). Risk factors of major depression in the elderly. European Psychiatry.

[11] Potvin O, Bergua V, Swendsen J, Meillon C, Tzourio C, Ritchie K, et al. Anxiety and 10-year risk of incident and recurrent depressive symptomatology in older adults. Depress Anxiety. 2013. 10.3389/fnins.2018.00248. 10.1002/da.22101 23532935

[12] Singh-Manoux Archana, Dugravot Aline, Ankri Joel, Nabi Hermann, Berr Claudine, Goldberg Marcel, Zins Marie, Kivimaki Mika, Elbaz Alexis (2014). Subjective cognitive complaints and mortality: Does the type of complaint matter?. Journal of Psychiatric Research.

[13] Rabin Laura A., Smart Colette M., Crane Paul K., Amariglio Rebecca E., Berman Lorin M., Boada Mercé, Buckley Rachel F., Chételat Gaël, Dubois Bruno, Ellis Kathryn A., Gifford Katherine A., Jefferson Angela L., Jessen Frank, Katz Mindy J., Lipton Richard B., Luck Tobias, Maruff Paul, Mielke Michelle M., Molinuevo José Luis, Naeem Farnia, Perrotin Audrey, Petersen Ronald C., Rami Lorena, Reisberg Barry, Rentz Dorene M., Riedel-Heller Steffi G., Risacher Shannon L., Rodriguez Octavio, Sachdev Perminder S., Saykin Andrew J., Slavin Melissa J., Snitz Beth E., Sperling Reisa A., Tandetnik Caroline, van der Flier Wiesje M., Wagner Michael, Wolfsgruber Steffen, Sikkes Sietske A.M. (2015). Subjective Cognitive Decline in Older Adults: An Overview of Self-Report Measures Used Across 19 International Research Studies. Journal of Alzheimer's Disease.

[14] Hertzog Christopher, Hülür Gizem, Gerstorf Denis, Pearman Ann M. (2018). Is subjective memory change in old age based on accurate monitoring of age-related memory change? Evidence from two longitudinal studies. Psychology and Aging.

[15] Hill NL, Mogle J, Whitaker EB, Gilmore-Bykovskyi A, Bhargava S, Bhang IY (2018). Sources of response bias in cognitive self-report items: “which memory are you talking about?” the gerontologist.

[16] Mogle Jacqueline, Hill Nikki, Bhang Iris, Bhargava Sakshi, Whitaker Emily, Kitt-Lewis Erin (2019). Time frame, problem specificity, and framing: the implicit structures of questions about memory in older adults. Aging & Mental Health.

[17] Hofer Scott M., Piccinin Andrea M. (2009). Integrative data analysis through coordination of measurement and analysis protocol across independent longitudinal studies. Psychological Methods.

[18] Kasper J, Freedman V (2018). National Health and Aging Trends Study (NHATS) user guide: rounds 1–7 beta release.

[19] Sonnega A., Faul J. D., Ofstedal M. B., Langa K. M., Phillips J. W., Weir D. R. (2014). Cohort Profile: the Health and Retirement Study (HRS). International Journal of Epidemiology.

[20] Kasper JD, Freedman VA, Spillman BC (2013). Classification of persons by dementia status in the National Health and aging trends study.

[21] McArdle JJ, Smith JP, Willis R. Cognition and economic outcomes in the health and retirement survey. Explor Econ Aging. 2011. 10.3386/w15266.

[22] Banks J, Muriel A, Smith JP. Attrition and health in ageing studies: evidence from ELSA and HRS. Longitud Life Course Stud. 2011. 10.14301/llcs.v2i2.115. 10.14301/llcs.v2i2.115 PMC3872999 24376472

[23] Chatfield Mark D., Brayne Carol E., Matthews Fiona E. (2005). A systematic literature review of attrition between waves in longitudinal studies in the elderly shows a consistent pattern of dropout between differing studies. Journal of Clinical Epidemiology.

[24] Kroenke Kurt, Spitzer Robert L., Williams Janet B. W. (2003). The Patient Health Questionnaire-2. Medical Care.

[25] Li Chunyu, Friedman Bruce, Conwell Yeates, Fiscella Kevin (2007). Validity of the Patient Health Questionnaire 2 (PHQ-2) in Identifying Major Depression in Older People. Journal of the American Geriatrics Society.

[26] Hox J (2010). Multilevel analysis: techniques and applications.

[27] Radloff Lenore Sawyer (1977). The CES-D Scale. Applied Psychological Measurement.

[28] Steffick DE, Wallace RB, Herzog AR, Ofstedal MB, Fonda S, Langa K. Documentation of affective functioning measures in the Health and Retirement Study. Univ Mich [Internet]. 2000; https://hrs.isr.umich.edu/sites/default/files/biblio/dr-005.pdf. Accessed 2 March 2019.

[29] Jonker Cees, Launer Lenore J., Hooijer Chris, Lindeboom Jaap (1996). Memory Complaints and Memory Impairment in Older Individuals. Journal of the American Geriatrics Society.

[30] MOJTABAI RAMIN, OLFSON MARK (2004). Major depression in community-dwelling middle-aged and older adults: prevalence and 2- and 4-year follow-up symptoms. Psychological Medicine.

[31] Elliot A. J., Mooney C. J., Douthit K. Z., Lynch M. F. (2013). Predictors of Older Adults' Technology Use and Its Relationship to Depressive Symptoms and Well-being. The Journals of Gerontology Series B: Psychological Sciences and Social Sciences.

[32] Sliwinski M, Buschke H. Cross-sectional and longitudinal relationships among age, cognition, and processing speed. Psychol Aging. 1999. 10.1037//0882-7974.14.1.18. 10.1037//0882-7974.14.1.18 10224629

[33] Lindenberger U, Baltes PB. Intellectual functioning in old and very old age: cross-sectional results from the Berlin aging study. Psychol Aging. 1997. 10.1037//0882-7974.12.3.410. 10.1037//0882-7974.12.3.410 9308090

[34] Nyberg L., Backman L., Erngrund K., Olofsson U., Nilsson L.-G. (1996). Age Differences in Episodic Memory, Semantic Memory, and Priming: Relationships to Demographic, Intellectual, and Biological Factors. The Journals of Gerontology Series B: Psychological Sciences and Social Sciences.

[35] Fiske Amy, Wetherell Julie Loebach, Gatz Margaret (2009). Depression in Older Adults. Annual Review of Clinical Psychology.

[36] Kinzer Adrianna, Suhr Julie A. (2015). Dementia worry and its relationship to dementia exposure, psychological factors, and subjective memory concerns. Applied Neuropsychology: Adult.

[37] Byers Amy L., Yaffe Kristine (2011). Depression and risk of developing dementia. Nature Reviews Neurology.

[38] Zimprich Daniel, Martin Mike, Kliegel Matthias (2003). Subjective Cognitive Complaints, Memory Performance, and Depressive Affect In Old Age: A Change-Oriented Approach. The International Journal of Aging and Human Development.

[39] Yoon Jong-Sung, Charness Neil, Boot Walter R, Czaja Sara J, Rogers Wendy A (2017). Depressive Symptoms as a Predictor of Memory Complaints in the PRISM Sample. The Journals of Gerontology: Series B.

[40] Weaver Cargin J., Collie A., Masters C., Maruff P. (2008). The nature of cognitive complaints in healthy older adults with and without objective memory decline. Journal of Clinical and Experimental Neuropsychology.

[41] Farrin Lydia, Hull Lisa, Unwin Catherine, Wykes Til, David Anthony (2003). Effects of Depressed Mood on Objective and Subjective Measures of Attention. The Journal of Neuropsychiatry and Clinical Neurosciences.

[42] Donaldson Catherine, Lam Dominic, Mathews Andrew (2007). Rumination and attention in major depression. Behaviour Research and Therapy.

[43] Rock P. L., Roiser J. P., Riedel W. J., Blackwell A. D. (2013). Cognitive impairment in depression: a systematic review and meta-analysis. Psychological Medicine.

[44] Whitfield K. E., Allaire J. C., Belue R., Edwards C. L. (2008). Are Comparisons the Answer to Understanding Behavioral Aspects of Aging in Racial and Ethnic Groups?. The Journals of Gerontology Series B: Psychological Sciences and Social Sciences.

[45] Potter Guy G., Steffens David C. (2007). Contribution of Depression to Cognitive Impairment and Dementia in Older Adults. The Neurologist.

[46] Folstein Marshal F., Folstein Susan E., McHugh Paul R. (1975). “Mini-mental state”. Journal of Psychiatric Research.

[47] Ciesielska Natalia, Sokołowski Remigiusz, Mazur Ewelina, Podhorecka Marta, Polak-Szabela Anna, Kędziora-Kornatowska Kornelia (2016). Is the Montreal Cognitive Assessment (MoCA) test better suited than the Mini-Mental State Examination (MMSE) in mild cognitive impairment (MCI) detection among people aged over 60? Meta-analysis. Psychiatria Polska.

[48] Rönnlund Michael, Sundström Anna, Adolfsson Rolf, Nilsson Lars-Göran (2015). Subjective memory impairment in older adults predicts future dementia independent of baseline memory performance: Evidence from the Betula prospective cohort study. Alzheimer's & Dementia.

[49] Wang Li, Van Belle Gerald, Crane Paul K., Kukull Walter A., Bowen James D., McCormick Wayne C., Larson Eric B. (2004). Subjective Memory Deterioration and Future Dementia in People Aged 65 and Older. Journal of the American Geriatrics Society.

[50] Schweizer S., Kievit R. A., Emery T., Henson R. N. (2017). Symptoms of depression in a large healthy population cohort are related to subjective memory complaints and memory performance in negative contexts. Psychological Medicine.

