# Supplementary material for: Memory complaints and depressive symptoms over time: a construct-level replication analysis
Source: BMC Geriatr. 2020 Mar 2;20:57. doi: 10.1186/s12877-020-1451-1 (PMC7050122; doi:10.1186/s12877-020-1451-1)
Supplement: Supplementary file 1 — Additional file 1: Table S1. NHATS: Baseline Mean Level Differences in Key Study Variables by Participants’ Age, Sex, Education, Race, Income, and Marital Status. [file 12877_2020_1451_MOESM1_ESM.docx]

**Supplementary Table**

Table 1. *NHATS*: *Baseline Mean Level Differences in Key Study Variables by Participants’ Age, Sex, Education, Race, Income, and Marital Status.*

|  | **Total Sample** | **Self-Rated Present Memory** | **Perceived two-year memory decline (Yes)** | **Depressive Symptoms** |
| --- | --- | --- | --- | --- |
|  | [%(n)] | [M(SD)] | [%(n)] | [M(SD)] |
| **Age differences** |  |  |  |  |
| 65-69^1^ | 28.35 (1437) | 2.38 (0.92)^4,5^ | 8.84 (127) | 2.82 (1.29) |
| 70-74^2^ | 25.55 (1295) | 2.41 (0.90) | 8.73 (113) | 2.74 (1.21) |
| 75-79^3^ | 19.39 (983) | 2.50 (0.89) | 9.87 (97) | 2.75 (1.14) |
| 80-84^4^ | 15.58 (790) | 2.52 (0.91)^1^ | 8.23 (65) | 2.71 (1.13) |
| 85-89^5^ | 7.40 (375) | 2.56 (0.94)^1^ | 12.53 (47) | 2.78 (1.27) |
| 90+^6^ | 3.73 (189) | 2.60 (0.89) | 11.64 (22) | 2.66 (1.09) |
| Significance Test |  | *F* (5) = 5.86 | χ^2^(5) = 8.20 | *F* (5) = 1.32 |
|  |  | *p* < .001, *η2 =* .01 | *p* = 0.14 | *p* = 0.25, *η2 =* .00 |
| **Sex differences** |  |  |  |  |
| Female | 63.64 (3226) | 2.47 (0.88) | 9.49 (306) | 2.82 (1.24) |
| Male_ref_ | 36.36 (1843) | 2.43 (0.95) | 8.95 (165) | 2.66 (1.16) |
| Significance Test |  | *Z = -2.30* | χ^2^(1) = 0.40 | *Z = - 5.84* |
|  |  | *p = 0.02, d = 0.05* | *p* = .53 | *p* < .001, d = 0.14 |
| **Education** |  |  |  |  |
| Less than High School^1^ | 13.03 (660) | 2.69^2,3^ (0.97) | 10.15 (67) | 3.06^2,3^ (1.45) |
| High School^2^ | 28.44 (1441) | 2.56^1,3^ (0.90) | 9.16 (28.03) | 2.89^1,3^ (1.28) |
| More than High School^3^ | 58.54 (2966) | 2.35^1,2^ (0.89) | 9.17 (272) | 2.63^1,2^ (1.09) |
|  |  |  |  |  |
| Significance Test |  | *F*(2,5061) = 51.05, | χ^2^(2 = 0.66, | *F*(2,5043) = 46.17, |
|  |  | *p* <.001, *η*^2^ = .02 | *p* = .72 | *p* <.001, *η*^2^ = .02 |
| **Race differences** |  |  |  |  |
| White | 78.77 (3993) | 2.40 (0.89) | 8.84 (353) | 2.70 (1.16) |
| Black | 21.23 (1076) | 2.66 (0.96) | 10.97 (118) | 2.98 (1.35) |
| Significance Test |  | *Z = 8.13* | χ^2^(1) = 4.55 | *Z = 6.53* |
|  |  | *p < .001, d = 0.29* | *p* = .03 | *p < .001, d = 0.22* |
| **Income level differences** |  |  |  |  |
| Below $15,000^1^ | 51.77 (2624) | 2.54 (0.93)^3,4^ | 10.63 (279) | 2.83 (1.26)^4^ |
| $15,000- $30,000^2^ | 13.55 (687) | 2.56 (0.92)^3,4^ | 8.15 (56) | 2.92 (1.33)^3,4^ |
| $30,000-$60,000^3^ | 16.95 (859) | 2.37 (0.87)^1,2,4^ | 7.33 (63) | 2.70 (1.16)^2,4^ |
| >$60,000^4^ | 17.74 (899) | 2.21 (0.84)^1,2,3^ | 8.12 (73) | 2.48 (0.92)^1,2,3^ |
| Significance Test |  | *F*(3,5062) = 36.21, | χ^2^(3) = 12.03, | *F*(3, 5044) = 24.54, |
|  |  | *p* <.001, *η*^2^ = .02 | *p* = .007 | *p* < .001, *η*^2^ =.01 |
| **Marital Status Differences** |  |  |  |  |
| Married/Partnered | 48.08 (2436) | 2.36 (0.88) | 8.42 (205) | 2.61 (1.08) |
| Separated/Divorced/Never Married | 51.92 (2631) | 2.54 (0.93) | 10.07 (265) | 2.90 (1.31) |
| Significance Test |  | *Z =* -7.25 | χ^2^(1) = 4.13 | *Z* = -8.97 |
|  |  | *p* < .001, *d* = 0.20 | *p* = .04 | *p* < .001, *d* = 0.25 |

*Note.* Superscripts represent group differences at  *p* < .01.
