# Supplementary material for: Memory complaints and depressive symptoms over time: a construct-level replication analysis
Source: BMC Geriatr. 2020 Mar 2;20:57. doi: 10.1186/s12877-020-1451-1 (PMC7050122; doi:10.1186/s12877-020-1451-1)
Supplement: Supplementary file 2 — Additional file 2: Table S2. HRS: Baseline Mean Level Differences in Key Study Variables by Participants’ Age, Sex, Education, Race, Income, and Marital Status. [file 12877_2020_1451_MOESM2_ESM.docx]

**Supplementary Table**

Table 2. *HRS*: *Baseline Mean Level Differences in Key Study Variables by Participants’ Age, Sex, Education, Race, Income, and Marital Status.*

|  | **Total Sample** | **Self-Rated Present Memory** | **Perceived two-year memory decline (Yes)** | **Depressive Symptoms** |
| --- | --- | --- | --- | --- |
|  | [%(n)] | [M(SD)] | [%(n)] | [M(SD)] |
| **Age (categorical)** |  |  |  |  |
| 65-69^1^ | 5.58 (307) | 2.798 (0.969)^4^ | 13.03 (40) | 1.329 (1.951)^5^ |
| 70-74^2^ | 28.90 (1591) | 2.896 (0.926)^4^ | 15.84 (252) | 1.180 (1.744)^4,5,6^ |
| 75-79^3^ | 31.79 (1750) | 2.922 (0.974) | 18.80 (329) | 1.327 (1.830)^4,5^ |
| 80-84^4^ | 20.38 (1122) | 3.070 (1.013)^1,2,3^ | 24.24 (272) | 1.611 (1.943)^2,3^ |
| 85-89^5^ | 9.97 (549) | 3.022 (1.041) | 23.13 (128) | 1.811 (2.096)^1,2,3^ |
| 90+^6^ | 3.38 (186) | 2.887 (1.126) | 29.57 (55) | 1.769 (1.873)^2^ |
|  |  | *F*(5,5449) = 6.81 | χ^2^(5) = 55.393 | *F*(5,5449) = 14.67 |
|  |  | *p* <.001, *η*^2^ = .01 | *p* < .001 | *p* <.001, *η*^2^ = .01 |
| **Sex differences** |  |  |  |  |
| Female | 64.78 (3566) | 2.911 (0.967) | 19.49 (695) | 1.544 (1.964) |
| Male | 35.22 (1949) | 3.0129 (1.009) | 19.65 (381) | 1.151 (1.678) |
| Significance Test |  | *z* = 4.011 | χ^2^(1) = 0.018, | *z* = -7.109 |
|  |  | *p* < .001, *d* = .103 | *p* = .893 | *p* < .001*,* *d* = .215 |
| **Education** |  |  |  |  |
| Less than High School^1^ | 38.07 (2096) | 3.111 (0.992)^2,3^ | 20.27 (425) | 1.770 (2.039)^2,3,4^ |
| High School^2^ | 47.30 (2604) | 2.881 (0.959)^1,3^ | 18.63 (485) | 1.264 (1.807)^1,4^ |
| More than High School^4^ | 14.62 (805) | 2.735 (0.975)^1,2^ | 20.62 (166) | 0.914 (1.440)^1,2^ |
|  |  | *F*(2,5502) = 54.66, | χ^2^(2) = 2.689, | *F*(2,5502) = 76.50, |
|  |  | *p* <.001, *η*^2^ = .02 | *p* = .261 | *p* <.001, *η*^2^ = .03 |
| **Race differences** |  |  |  |  |
| White | 89.65 (4935) | 2.930 (.971) | 18.99 (937) | 1.374 (1.857) |
| Black | 10.35 (570) | 3.095 (1.071) | 24.39 (139) | 1.677 (2.030) |
| Significance Test |  | *z* = 3.624 | χ^2^(1) = 9.4722, | *z* = 3.794 |
|  |  | *p* < .001, *d* = .161 | *p* = .002 | *p* = .001, *d* = .156 |
| **Income level differences** |  |  |  |  |
| Below $15,000^1^ | 36.60 (2015) | 3.019 (1.026)^3,4^ | 20.44 (412) | 1.884 (2.096)^2.3,4^ |
| $15,000- $30,000^2^ | 33.41 (1839) | 2.954 (0.941)^4^ | 18.65 (343) | 1.270 (1.761)^1,3,4^ |
| $30,000-$60,000^3^ | 20.02 (1102) | 2.868 (0.944)^1^ | 18.78 (207) | 1.011 (1.607)^1,2^ |
| >$60,000^4^ | 9.97 (549) | 2.818 (1.020)^1,2^ | 20.77 (114) | .894 (1.482)^1,2^ |
| Significance Test |  | *F*(3,5501) = 9.22, | χ^2^(3) = 2.901 | *F*(3, 5501) = 79.85, |
|  |  | *p* <.001, *η*^2^ = .01 | *p* = .407 | *p* < .0001, *η*^2^ =.04 |
| **Marital Status Differences** |  |  |  |  |
| Married/Partnered | 52.16 (2633) | 2.2963 (0.961) | 19.68 (565) | 1.107 (1.674) |
| Separated/Divorced/Never Married | 47.84 (2871) | 2.929 (1.007) | 19.37 (510) | 1.730 (2.027) |
|  |  | *z* = -1.105 | χ^2^(1) = .084 | *z* = 12.834 |
|  |  | *p =* .269*, d =* .653 | *p =* .772 | *p <* .001*, d* = .335 |

*Note.* Superscripts represent group differences at *p* < .01.
